# Model Establishment of a Co-Based Metal Matrix with Additives of WC and Ni by Discrete Element Method

**DOI:** 10.3390/ma11112319

**Published:** 2018-11-19

**Authors:** Xiuyu Chen, Guoqin Huang, Yuanqiang Tan, Hui Huang, Hua Guo, Xipeng Xu

**Affiliations:** 1 Institute of Manufacturing Engineering, Huaqiao University, Xiamen 361021, China; yu@hqu.edu.cn (X.C.); tanyq@hqu.edu.cn (Y.T.); huangh@hqu.edu.cn (H.H.); guoh1214@hqu.edu.cn (H.G.); xpxu@hqu.edu.cn (X.X.); 2 Fujian Engineering Research Center of Intelligent Manufacturing for Brittle Materials, Huaqiao University, Xiamen 361021, China; 3 MOE Engineering Research Center for Machining of Brittle Materials, Huaqiao University, Xiamen 361021, China

**Keywords:** metal-bonded diamond tools, metal matrix, discrete element method (DEM), microcosmic parameters, transverse rupture strength (TRS)

## Abstract

A metal matrix is an indispensable component of metal-bonded diamond tools. The composition design of a metal matrix involves a number of experiments, making costly in terms of time, labor, and expense. The discrete element method (DEM) is a potential way to relieve these costs. The aim of this work is to demonstrate a methodology for establishing and calibrating metal matrix’s DEM model. A Co-based metal matrix with WC and Ni additives (CoX–WC–Ni) was used, in which the Co-based metal was Co–Cu–Sn metal (CoX). The skeletal substances in the metal matrix were treated as particles in the model, and the bonding substances were represented by the parallel bond between particles. To describe the elasticity of the metal matrix, a contact bond was also loaded between particles. A step-by-step calibration procedure with experimental tests of three-point bending and compression was proposed to calibrate all microcosmic parameters involved during the establishment of DEM models: first for the CoX matrix, then for the CoX–WC matrix and CoX–Ni matrix, and finally for the CoX–WC–Ni matrix. The CoX–WC–Ni DEM model was validated by the transverse rupture strength (TRS) of two new compositions and the results indicated that the model exhibited a satisfactory prediction ability with an error rate of less than 10%.

## 1. Introduction

A metal matrix is essential for the metal matrix diamond segment, which is an indispensable component of metal-bonded diamond tools [1]. The wear performance of a metal-bonded diamond tool mainly depends on the property of its metal matrix, in which diamonds are embedded through solid–liquid sintering [1,2,3,4]. The primary role of the metal matrix is to keep the diamonds in service for as long as possible. In general, a metal matrix is a typical multiple and mutative material because it is composed of more than two metal ingredients [5]. A change in the combinations of metal ingredients and their contents will result in greatly different matrix performance. Therefore, during the design of a metal matrix, when the metal ingredients and their contents are changed, the number of required performance confirmation experiments would significantly increase and the costs of time and labor would be greatly increased as a result [6].

Recently, the using of numerical simulations has attracted great attention due to its potentiality in lowing cost involved in the design of a metal matrix. Several scholars used the finite element method (FEM) to simulate the residual stress and retention of metal matrix [7,8,9]. In these cases, the metal matrix was treated as a continuous, unified elastic–plastic material with combined isotropic–kinematic hardening. However, the metal matrix of the diamond segment is not just a kind of multi-component and discontinuous material, but more likes a brittle material [10]. In view of this, the Discrete Element Method (DEM) might be more properly to describe this material. DEM was developed by Cundall and Strack [11] to simulate the behaviors of various non-continuous and non-homogeneous materials, such as granular [12,13,14], silicon nitride ceramics [15], concrete materials [16], and nanomaterials [17,18,19]. Our recent work proved that DEM was useful for investigating the transverse rupture strength (TRS) of metal matrix diamond segment [20].

However, during establishing the block model by DEM, the calibration of the microcosmic parameters of the particles in the models is vital, as these parameters directly determine the macro–physical and macro–mechanical parameters of the DEM simulation. A number of studies have been devoted to obtaining these parameters [10,14,20,21,22]; however, to our knowledge, few studies were focused on the calibrations of the microcosmic parameters involved in the DEM model of the metal-bonded diamond tools’ metal matrix with metal additives.

The objective of this work is to demonstrate the establishment of the metal matrix’s DEM model and its related microcosmic parameters’ calibration. A Co-based metal matrix with Ni and WC metal additives (CoX–WC–Ni) was used in this work because it is widely used in metal-bonded diamond tools [23]. The methodology of the established DEM model and the calibration of its relevant microcosmic parameters are described and analyzed in detail. The transverse rupture strength (TRS) of the metal matrix is used for validating the built DEM model by comparing it with the corresponding experimental and simulated results.

## 2. Metal Matrix Discrete Element Method (DEM) Model Establishment and Calibration

**Figure 1:**
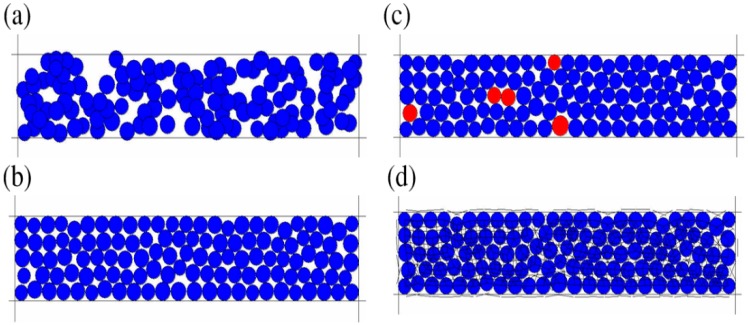
Steps for building material block in discrete element method (DEM) model: (**a**) ball-particles generated, (**b**) ball-particles balanced, (**c**) floating particles deleted, and (**d**) material block formed by bonding.

The DEM simulation was implemented using the software of PFC^2D^ (Particle Flow Codes in two dimensions, Itasca Consulting Group Inc., Version 3.1). Generally, as shown in Figure 1, it takes four steps to build a DEM model, which are: (a) generating different kinds of ball-particles with a defined range of radii in the range with a defined length and a defined width; (b) balancing all of the ball-particles according to some initial stress; (c) deleting floating ball-particles; (d) giving the bonding values of each microcosmic parameters of the particles and bonds in the model to make the ball-particles into a material block with defined mechanical properties.

According to the above four steps, there are three crucial points for the construction of the DEM model: the accumulation of ball-particles, the bonds among particles that bond the particles to accumulate to form a block, and the microcosmic parameters of bonds that describe the mechanical behaviors of the block. To build a DEM model for a Co-based metal matrix of diamond segment, these three points are considered below.

### 2.1. Metal Matrix

**Figure 2:**
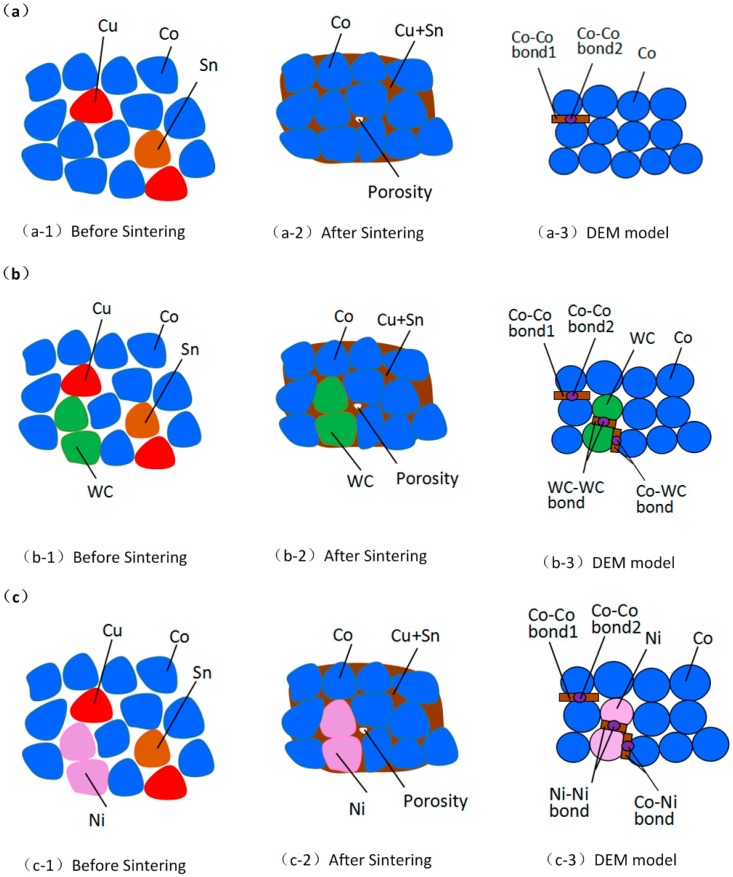

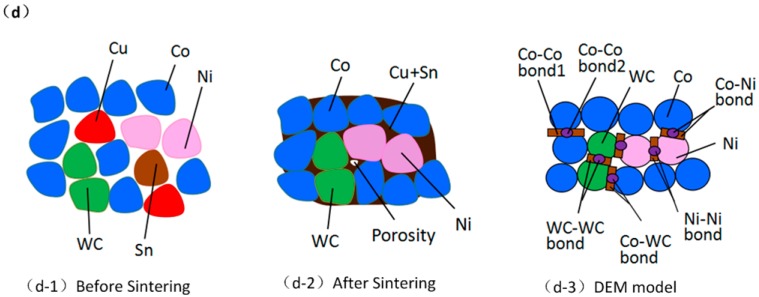
The status and model of the metal matrix with different compositions: (**a**) CoX matrix, (**b**) CoX–WC matrix, (**c**) CoX–Ni matrix, and (**d**) CoX–WC–Ni matrix.

The metal matrix of diamond segments is prepared by a solid–liquid sintering process under heat and pressure. Generally, the metals in a metal matrix can be divided into two classes: (i) metal powders with high melting temperatures, such as Co, WC, Ni and Ti [5,24], which are not melted during the sintering process and act as a skeletal substance in the matrix; and (ii) metal powders with low melting temperature, such as Cu, Sn and Zn, which are melted into liquid phases during sintering and act as a bond substance. In this work, to simplify the DEM model, the particles in the model only denote the skeletal substance, while the bonding substance is represented by the parallel bond between particles [25], which will be described below. As the basic component of the Co-based matrix used in this work was Co–Cu–Sn (shortened to CoX), it is clear that Co is the skeletal metal and Cu–Sn serves as the bonding agent. Therefore, all particles in the DEM model of CoX are Co, as shown in Figure 2a. In addition, as the skeletal substances do not melt during sintering, pores inevitably exist inside the matrix. We set the porosity rate to 0.08 in the model according to our previous real measurement.

Commonly, Ni and WC are added into CoX for the aim of improving the matrix’s wear resistance. Due to their high melting temperatures, Ni and WC also act as skeletal substance. That means some of the particles in the DEM model of the CoX were replaced by Ni and WC, as shown in Figure 2b–d. As a result, the DEM model of a CoX matrix with other additives was formed.

**Figure 3:**
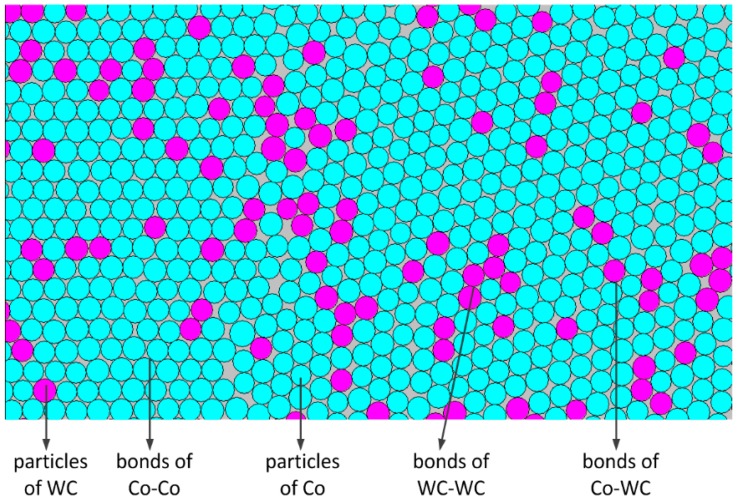
Particles in the DEM model of the CoX–WC.

In the present work, the DEM models were built with dimensions of 30 mm × 6 mm for three-point bending tests and 6 mm × 6 mm for compression tests. In the model, the radius of the particles was 24 µm. The number of particles in a 30 mm × 6 mm area was 87,852, and that in a 6 mm × 6 mm area was 17,570. To build a DEM model for CoX with additives, new additive particles are used instead of the original Co particles and the additive particles were randomly disturbed in the model. Figure 3 shows the DEM model of CoX–WC. Co particles were replaced by WC particles. The number of replacements depended on the additive’s content, as listed in Table 1.

### 2.2. Bonds in the Metal Matrix Model

**Figure 4:**
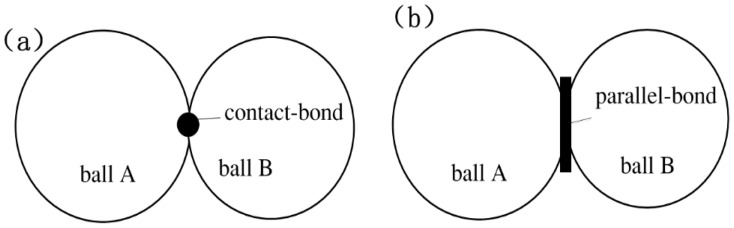
Demonstration of (**a**) contact bond and (**b**) parallel bond.

In the DEM model of a metal matrix, as mentioned above, the word ‘particles’ is used to denote the skeletal substances, while ‘bond’ is used to describe the bonding substances. A typical microstructure of bonded materials consists of grains cemented by a bond material at the contacts [16]. As the metal matrix is sintered by pressing under hot temperature, metal particles squeeze each other and the adjacent particles are deformed. A contact bond in the DEM model is considered to be the interaction between two grains since a grain itself is deformable. The contact bond is related to the elastoplasticity of the material. A parallel bond is considered to represent the physical behavior of a bond substance among bonded particles [26]. In DEM models of typical brittle materials, such as granular, silicon nitride ceramics, and concrete, parallel bonds exist alone [9,10,11,12,13,14]. However, the stress-strain curves of the metal matrix indicate that the metal matrix simultaneously has a certain elasticity and brittleness [16]. The metal matrix is first subjected to plastic deformation and then to fracture failure in brittle mode. Owing to this fact, in this work, both parallel bonds and contact bonds are used to describe the contact boundary of every bond pair of particles in the DEM model of a Co-based matrix, as shown in Figure 4. Therefore, all possible bond pairs of particle bonds of the CoX matrix with Ni and WC additions are summarized in Table 2.

From Figure 2 and Table 2, it can be seen that there are four different combinations of CoX with Ni and WC additives, and the details of their corresponding DEM model are different, detailed as follows:

1. In the DEM model of the CoX matrix, which is without any additives, the bonded pair is only the Co–Co pair. The bond boundary of Co‒Co includes Co–Co bond1 and Co–Co bond2, which are a parallel bond and contact bond, respectively, as shown in Figure 4.
2. In the DEM model of CoX–WC, the addition of WC particles introduces two new bonded pairs: WC–WC and Co–WC.
3. In the DEM model of CoX–Ni, the addition of Ni particles introduces two new bonded pairs: Ni–Ni and Co–Ni.
4. In the DEM model of CoX–WC–Ni, the simultaneous addition of WC and Ni introduces a new bonded pair: Ni–WC.

The number of bonds depends upon the number and distribution of particles in the DEM model. The number of WC or Ni particles is related to the proportion, but their distributions are random. Table 1 shows the numbers of bonded pairs in the DEM matrix with different proportions of WC addition.

### 2.3. Microcosmic Parameters

#### 2.3.1. Inversion Method for Microcosmic Parameters

The macrocosmic parameters of the physical and mechanical characteristics for a medium cannot be assigned directly in a DEM model, while the geometric characteristic parameters of particles, i.e., their microcosmic parameters and those of the bonds between particles, can. However, microcosmic parameters of particles and bonds cannot be obtained directly, except for the density of a particle. As the macro-physical and macro-mechanical parameters depend upon these microcosmic parameters, these parameters are commonly calibrated by comparing the macrocosmic outcome of large-scale DEM simulations with bulk attempts [14,18,27]. This method is called inversion.

**Figure 5:**
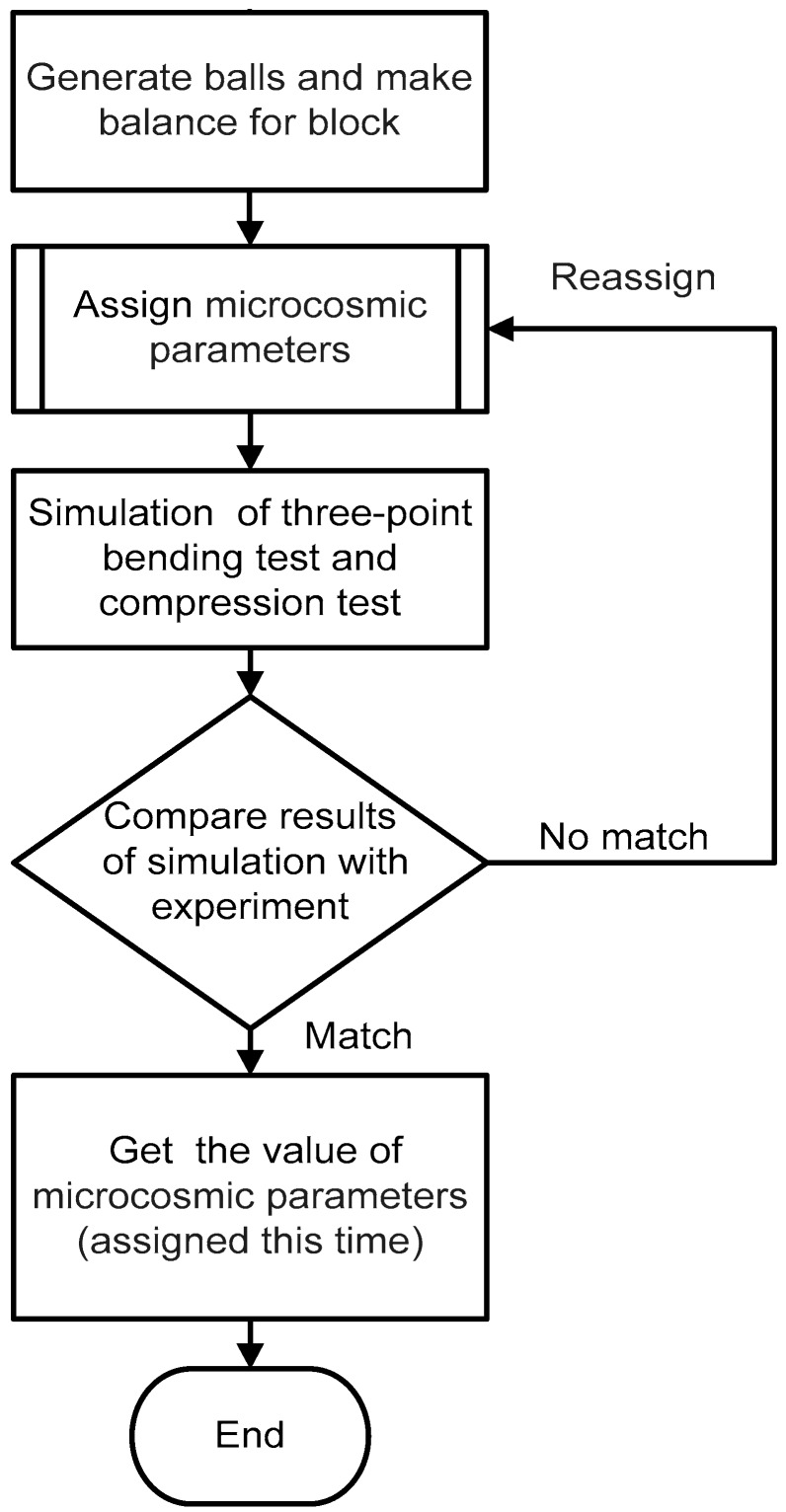
Inversion procedure.

**Figure 6:**
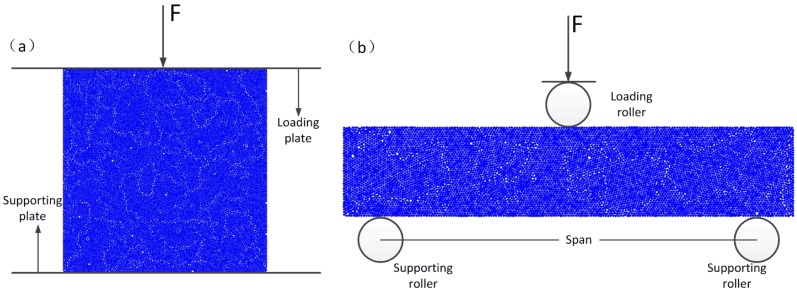
DEM modeling of (**a**) compression test and (**b**) three-point bending test.

The inversion procedure is shown in Figure 5. A model is built with the given geometric parameters as an experimental sample, and each microcosmic parameter in the model was assigned. Simulations of the three-point bending and compression tests are carried out, as shown in Figure 6, to obtain the values of transverse rupture strength (TRS), uniaxial compressive strength (UCS), and Young’s modulus (Ec) and compare them with the corresponding experimental results. When the simulated results match the experimental results with an error of less than 10%, the microcosmic parameters of the DEM model are considered to be calibrated.

#### 2.3.2. Calibration of Microcosmic Parameters for CoX–WC–Ni

Owing to the fact that the metal matrix of diamond segments has more than one kind of metal component, its model involves more microcosmic parameters than those of other single-substance materials. For CoX–WC–Ni, there are three particle types and six bonded pairs among the particles in the DEM model, as shown in Table 2. The molecular dynamic simulations were proposed to be a method to calibrate contacts in carbon nanotubes, gold, and CdSe nanoparticles which were in nanoscale [25,26,27], but it is not applicable to the metal matrix because of the scale problem.

**Figure 7:**
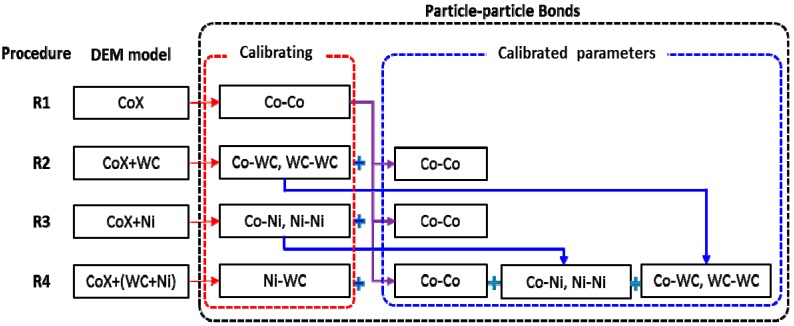
Step-by-step procedure for calibrating bonds’ microcosmic parameters.

The other major difficulty is to obtain all the microcosmic parameters of these particles and bonds simultaneously. In this paper, a step-by-step calibration method was proposed to reduce this difficulty. To carry this out, four calibration procedures are summarized in Figure 7 and detailed below:

R1: obtaining the microcosmic parameters of Co particles and Co–Co bonds. With the DEM model of the CoX matrix, the microcosmic parameters of Co particles and the Co–Co bonds were inverted.

R2: obtaining microcosmic parameters of WC particles, WC–WC and Co–WC bonds. As shown in Figure 2, the bonds in the DEM model of the CoX–WC matrix are Co–Co, WC–WC, and Co–WC. The microcosmic parameters of Co particles and the Co–Co bonds are set based on the results of R1. Then, the microcosmic parameters of the WC particles, WC–WC, and Co–WC bonds are inverted.

R3: obtaining microcosmic parameters of Ni particles, Ni–Ni, and Co–Ni bonds. Referring to R2, the microcosmic parameters of the Ni particles, Co–Ni, and Ni–Ni bonds are inverted by the DEM model of CoX–Ni.

R4: obtaining microcosmic parameters of Ni–WC bonds. Based on the results of R1–R3, the microcosmic parameters of Ni–WC are inverted by the DEM model of CoX–Ni–WC.

With these four procedures, the microcosmic parameters of all particles and bonds in the DEM model of CoX–Ni–WC are achieved.

## 3. Experimental Setup

### 3.1. Metal Matrix Composition

A Co70–Cu27–Sn3 (wt %) composition was used as the CoX matrix, and powders of WC and Ni were used as additives. The original particle sizes and purities of metals used are listed in Table 3.

In accord with the microcosmic parameter calibration procedure presented above, six Co-based matrix compositions were designed and detailed in Table 4. For the validation, two compositions, namely CoX90–WC5–Ni5 and CoX80–WC10–Ni10, were designed and also listed in Table 4.

### 3.2. Fabrication of Metal Matrix

For every composition of metal matrix, metal powders were mixed together with a defined composition and blended for 120 min via a rotary mixer (SYH01, Chunlai Machinery, Changzhou, China), then poured into a graphite mold, and finally sintered at a temperature of 810 °C with a hold time of 2 min and a hold pressure of 15 MPa on an automatic hot-pressing sinter machine (SMVBC, Golden Highway, Zhengzhou, China), which was installed with an infrared sensor (RS-WD-HW-120, Jiandakeren, Shanghai, China) for temperature monitoring. After being sintered and cooled, the metal matrix specimen was obtained from the mold.

### 3.3. Three-Point Bending Test and Compression Test

Metal matrices with dimensions of 30 mm × 12 mm × 6 mm were fabricated for three-point bending tests and dimensions of 6 mm × 6 mm × 6 mm were fabricated for compression tests. The TRS and Young’s modulus (Ec) were determined by a three-point bending test in which the bending span was set to 25 mm. The uniaxial compressive strength (UCS) was determined by compression test. Both tests were performed on an Instron 5569 tester (Instron, Boston, MA, USA).

For each condition of test, 10 specimens were repeated to obtain the averaged value. A 3D digital microscope (KH8700, HIROX, Tokyo, Japan) was used to observe the fracture of the specimen after testing.

## 4. Calibration Results

**Figure 8:**
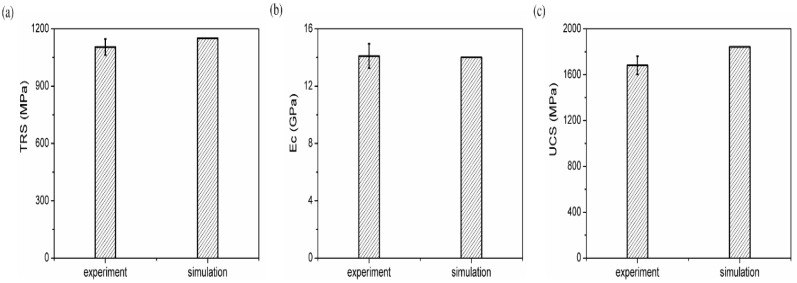
Comparisons between the experimental and the simulation results: (**a**) TRS, (**b**) Ec, and (**c**) UCS.

According to the calibration procedures presented in Section 2, the first step was to obtain the microcosmic parameters of Co particles and the Co–Co bonds, as in procedure R1, and the composition of matrix was CoX100, as listed in Table 4. After all the particles were generated, the microcosmic parameters were initially assigned for particle assembly; then, the values of TRS, UCS and Ec were obtained from the simulations of three-point bending and compression tests. The values of the microcosmic parameters were adjusted in order to make the simulation results to match their corresponding experimental results. After debugging the values repeatedly in accordance with the inversion procedure shown in Figure 5, the microcosmic parameters of Co particles and Co–Co bonds were achieved and listed in Table 5 and Table 6. The final calculated results (TRS, UCS, and Ec) and their experimental results are compared in Figure 8. It can be seen that the values of TRS, UCS, and Ec obtained from the simulation agreed well with their corresponding experimental results. As seen from Figure 8a, the average TRS of the experiment was 1120 MPa with a standard deviation of 38 MPa; the TRS of the simulation was 1150 MPa. Figure 8b shows that the average UCS of the experiment was 1681 MPa with a standard deviation of 80 MPa; the UCS of the simulation was 1780 MPa. Figure 8c shows that the average Ec of the experiment was 13.8 GPa with a standard deviation of 0.8 GPa; the Ec of the simulation was 13.7 GPa. The corresponding errors of TRS, UCS, and Ec were 2.6%, 6.0%, and, 0.7% respectively. All the errors were within 10%.

According to procedure R2, the microcosmic parameters of WC particle, Co–WC bonds and WC–WC bonds are inverted for composition CoX95–WC5. The results are listed in Table 5 and Table 6. In addition, with the above-calibrated microcosmic parameters, the other two compositions, CoX97–WC3 and CoX90–WC10, were also simulated and the results of TRS, and Ec are compared in Table 7. It can be seen that when all of the corresponding microcosmic parameters were determined, both adding WC to the basic model and changing the proportion of WC in the model changed the results of the simulation. Moreover, the simulation results are close to the experimental ones, which supports the assumption that microcosmic parameters for the bonds in the DEM model do not change with the matrix composition.

The composition CoX97–Ni3 was used for procedure R3, and the values of the related microcosmic parameters of Ni particles, Ni–Ni bonds and Co–Ni bonds were obtained as listed in Table 5 and Table 6, too.

Finally, the microcosmic parameters of the Ni–WC bonds were inverted according to procedure R4. The matrix composition used is CoX94–(WC3–Ni3). The results are listed in Table 6.

With the above calibrations, the microcosmic parameters of Co, WC, and Ni in the DEM model of CoX–WC–Ni were determined and summarized in Table 5, and the microcosmic parameters of the Co–Co, Co–Ni, Co–WC, WC–WC, Ni–Ni, and Ni–WC bonds were calibrated and summarized in Table 6.

## 5. Validation and Discussion

It is difficult to design the composition of a diamond–segment matrix because there too many combinations. An attempt is made to design the composition using DEM simulation. In the diamond–tool industry, TRS is an important performance indicator for determining the composition of the metal matrix of a diamond segment [19]. Even if the ingredients are the same, TRS differs when the proportions of each ingredient differ [21]. A DEM model with calibrated microcosmic bond parameters was constructed for this study. Then, the work was performed to validate the practicability of the model.

Using the CoX–WC–Ni DEM model, two new compositions of CoX90–(Ni5–WC5) and CoX80–(Ni10–WC10) were simulated. Their TRS results were consistent with the corresponding experimental results, as compared in Table 8. Both simulation error rates were less than 10%. This shows the correctness of the model built above.

## 6. Concluding Remarks

DEM models for a Co–Cu–Sn diamond metal matrix with additions of WC and Ni were established in this work. The microcosmic parameters of bonds in the model were calibrated. The practicality of the model was also proved by validation. Some notable observations for the proposed work are as follows:

1. When building the DEM model of the diamond metal matrix, skeletal substances in the matrix are treated as particles in the model and bonding substances can be represented as parallel bonds between particles.
2. Besides the parallel bond, the contact bond should be considered during the construction of the DEM model of the diamond segment matrix because of its elasticity.
3. The step-by-step calibration of microcosmic parameters is effective for the DEM model of metal matrix with multiple additives.
4. The constructed CoX–WC–Ni DEM model exhibited a satisfactory ability to predict TRS, and the error rate is less than 10%. It will be useful for the design of a metal matrix.

## Figures and Tables

**Table 1:** Number of WC particles and bonds in a sample of area 30 mm × 6 mm.

Percentage of WC	Number of WC Particles	WC–WC Bonds		WC–Co Bonds	
		Number	Proportion	Number	Proportion
10%	12,550	4959	1.9%	60,956	24.6%
5%	6275	1241	0.5%	32,929	13.3%
3%	3765	464	0.2%	20,317	8.2%

**Table 2:** Types of bonded pairs of particles in the DEM model of Co-based matrix.

Metal Matrix	Particles in the Model	Bonded Pairs between Particles
CoX	Co	Co–Co
CoX–WC	Co, WC	Co–Co, Co–WC, WC–WC
CoX–Ni	Co, Ni	Co–Co, Co–Ni, Ni–Ni
CoX–WC–Ni	Co, Ni, WC	Co–Co, Co–WC, WC–WC, Co–Ni, Ni–Ni, Ni–WC

**Table 3:** Size and purity of metal powders used to fabricate metal matrix specimens.

Ingredient	Average Granularity (µm)	Purity (%)
Cobalt (Co)	48	99.7
Copper (Cu)	48	99.5
Tin (Sn)	74	98.0
Nickel (Ni)	48	99.6
Tungsten carbide (WC)	48	99.9

**Table 4:** Compositions of metal matrix specimens.

No.	Matrix	CoX (wt %)	WC (wt %)	Ni (wt %)
1	CoX100	100	0	0
2	CoX97–WC3	97	3	0
3	CoX95–WC5	95	5	0
4	CoX90–WC10	90	10	0
5	CoX97–Ni3	97	0	3
6	CoX94–WC3–Ni3	94	3	3
7	CoX90–WC5–Ni5	90	5	5
8	CoX80–WC10–Ni10	80	10	10

**Table 5:** Microcosmic parameters of particles.

Particles	Particles		
	Co	WC	Ni
ρ, Density of particles, g/cm^3^	8.90	15.63	8.88
Ec, Elasticity modulus of particles, (Pa)	1.3 × 10^10^	8 × 10^11^	9 × 10^9^
μ, Friction coefficient	0.80	0.05	0.80
R_max_/R_min_ *	1.13	1.13	1.13

* R_max_/R_min_ is a coefficient about the radius range.

**Table 6:** Parameters of Co-based metal matrix bonds upon additions of WC and Ni.

Bonds	Microcosmic Parameters	Type of Particle Bonds					
		Co–Co	WC–WC	Co–WC	Ni–Ni	Co–Ni	Ni–WC
Contact bond	Normal strength (Pa)	6 × 10^7^	12 × 10^7^	10 × 10^7^	7 × 10^7^	7.5 × 10^7^	9.5 × 10^7^
	Shear strength (Pa)	6 × 10^7^	12 × 10^7^	10 × 10^7^	7 × 10^7^	7.5 × 10^7^	9.5 × 10^7^
Parallel bond	Elasticity modulus (Pa)	1.3 × 10^9^					
	Normal strength (Pa)	3 × 10^8^					
	Shear strength (Pa)	3 × 10^8^					
	Radius multiplier	1					

Note: The ratio of normal strength to shear strength in this work was set to 1.

**Table 7:** Comparison between experimental and simulation results for WC specimens.

The Content of WC	TRS			Ec			UCS		
	Experiment (MPa)	Simulation (MPa)	Error (%)	Experiment (GPa)	Simulation (GPa)	Error (%)	Experiment (MPa)	Simulation (MPa)	Error (%)
3%	1054 ± 85	1180	11.9	13.9 ± 0.9	15.3	10.1	1713 ± 82	1819	6.2
5%	1015 ± 79	1144	12.7	13.7 ± 0.7	14.2	3.7	1751 ± 77	1893	8.2
10%	967 ± 82	1038	7.3	13.4 ± 0.8	13.7	2.2	1825 ± 83	1965	7.7

**Table 8:** TRS values of simulation and experiment for different compositions.

TRS	CoX90–(WC5–Ni5)	CoX80–(WC10–Ni10)
Simulation	1110 MPa	981 MPa
Experimental	1035 MPa	923 MPa
Error rate	7.7%	5.9%
